# Sparse Evidence for *Giardia intestinalis*, *Cryptosporidium* spp. and Microsporidia Infections in Humans, Domesticated Animals and Wild Nonhuman Primates Sharing a Farm–Forest Mosaic Landscape in Western Uganda

**DOI:** 10.3390/pathogens10080933

**Published:** 2021-07-23

**Authors:** Marie Cibot, Matthew R. McLennan, Martin Kváč, Bohumil Sak, Caroline Asiimwe, Klára Petrželková

**Affiliations:** 1 Department of Social Sciences, Faculty of Humanities and Social Sciences, Oxford Brookes University, Oxford OX3 0BP, UK; mmclennan@brookes.ac.uk; 2 Bulindi Chimpanzee & Community Project, Hoima P.O. Box 245, Uganda; 3 Anicoon Vétérinaires, Ploemeur, 56260 Larmor-Plage, France; 4 Biology Centre, Institute of Parasitology, Czech Academy of Sciences, 370 05 České Budějovice, Czech Republic; kvac@paru.cas.cz (M.K.); casio@paru.cas.cz (B.S.); petrzelkova@ivb.cz (K.P.); 5 Faculty of Agriculture, University of South Bohemia, 370 05 České Budějovice, Czech Republic; 6 Budongo Conservation Field Station, Masindi P.O. Box 362, Uganda; asiimwecaroline@gmail.com; 7 Institute of Vertebrate Biology, Czech Academy of Sciences, 603 65 Brno, Czech Republic; 8 Liberec Zoo, 460 01 Liberec, Czech Republic

**Keywords:** *Giardia*, *Cryptosporidium*, microsporidia, non-human primates, humans, domestic animals, anthropogenic disturbance, coproantigen, PCR, Uganda

## Abstract

Zoonotic pathogen transmission is considered a leading threat to the survival of non-human primates and public health in shared landscapes. *Giardia* spp., *Cryptosporidium* spp. and Microsporidia are unicellular parasites spread by the fecal-oral route by environmentally resistant stages and can infect humans, livestock, and wildlife including non-human primates. Using immunoassay diagnostic kits and amplification/sequencing of the region of the triosephosphate isomerase, small ribosomal subunit rRNA and the internal transcribed spacer genes, we investigated *Giardia*, *Cryptosporidium*, and microsporidia infections, respectively, among humans, domesticated animals (livestock, poultry, and dogs), and wild nonhuman primates (eastern chimpanzees and black and white colobus monkeys) in Bulindi, Uganda, an area of remarkably high human–animal contact and spatial overlap. We analyzed 137 fecal samples and revealed the presence of *G. intestinalis* assemblage B in two human isolates, *G. intestinalis* assemblage E in one cow isolate, and *Encephalitozoon cuniculi* genotype II in two humans and one goat isolate. None of the chimpanzee and colobus monkey samples were positive for any of the screened parasites. Regular distribution of antiparasitic treatment in both humans and domestic animals in Bulindi could have reduced the occurrence of the screened parasites and decreased potential circulation of these pathogens among host species.

## 1. Introduction

Emerging zoonotic diseases are a serious threat to both public health and animal conservation. While emerging epidemics such as Ebola and more recently Severe acute respiratory syndrome coronavirus 2 (SARS-CoV-2), most likely resulting from zoonotic transmission, can be deadly among humans [1,2], lethal cases of other human respiratory outbreaks are also described in wild nonhuman primates (NHP), particularly great apes [3,4]. Cross-species transmission of pathogens also represents a major threat to health and survival of wild NHP [5,6,7,8], whose populations are declining rapidly in many regions [9,10].

Human activities, including logging, forest clearance, and farming, have meant that NHP increasingly share anthropogenically modified landscapes with humans and livestock [11,12,13]. The increased spatial proximity between species enhances the risk of pathogen transmission [14,15,16]. Therefore, parasitological surveys of NHP and humans sharing habitats are of great interest for understanding the consequences of close human–NHP coexistence by identifying taxa with pathogenic and zoonotic potential [17,18].

*Giardia intestinalis*, *Cryptosporidium* spp., and microsporidia of genera *Encephalitozoon* and *Enterocytozoon* are common intestinal protists infecting humans and domesticated animals, including livestock, dogs, and cats [19,20,21,22]. These unicellular organisms also infect great apes and other NHP [23,24,25,26,27]. In humans, giardiosis and cryptosporidiosis are characterized by chronic diarrhea, abdominal cramps, and weight loss, and in immunodeficient hosts infections can be fatal [28,29]. In free-ranging and captive apes, both zoonotic assemblages A and B, and ungulate specific assemblages E of *G. intestinalis* have been reported, but infections were asymptomatic [23,30,31,32]. The study of *Cryptosporidium* infections in great apes is limited to a few studies. To date, several *Cryptosporidium* species have been described in great apes, namely *C. parvum* in mountain gorillas (*Gorilla beringei beringei*) and Bornean orangutans (*Pongo pygmaeus*), *C. muris* in western lowland gorillas (*G. gorilla gorilla*), Bornean orangutans and Sumatran orangutans (*Pongo abelii*), *C. bovis* and *C. meleagridis* in western lowland gorillas, and *C. hominis*, *C. suis*, and *C. ubiquitum* in eastern chimpanzees (*Pan troglodytes schweinfurthii*) [7,26,27,30,33,34,35,36]. Additionally, *C. parvum*, *C. hominis*, *C. hominis* “monkey”, *C. felis*, and *C. cuniculus* have been reported from other NHPs such as macaques, langurs, colobus, and baboons [37,38,39,40,41]. In agreement with studies conducted in humans, many infections in apes were subclinical [25,26,27,30]. The abnormal appearance of the stools of gorillas (i.e., presence of blood and mucus) was observed in a few cases in animals with the highest values of oocyst concentration [42]. Microsporidial infections caused by *Encephalitozoon* spp. and *E. bieneusi* in humans and NHP are characterized by a variety of pathologies ranging from asymptomatic to lethal infections, mainly in immunodeficient hosts [43]. In contrast to other NHP [44,45,46], clinical disease or pathological findings have not been reported in apes. The clinical impact is unknown, but it is suggested that the course of infection is similar to that in humans.

As *Giardia*, *Cryptosporidium*, and microsporidia infections result from fecal-oral transmission through ingestion of contaminated water and food, transmission might occur between humans, domestic animals, and wildlife sharing environments [47,48]. Previous studies that explored genetic diversity of *G. intestinalis*, *Cryptosporidium* spp., and microsporidia in wild great apes revealed potential for transmission of those parasites among humans, domestic animals, and mountain gorillas in Uganda [34,35,42,49,50] and Rwanda [27,51], and western gorillas in Central African Republic [26], and within orangutan populations on Sumatra [30].

**Figure 1:**
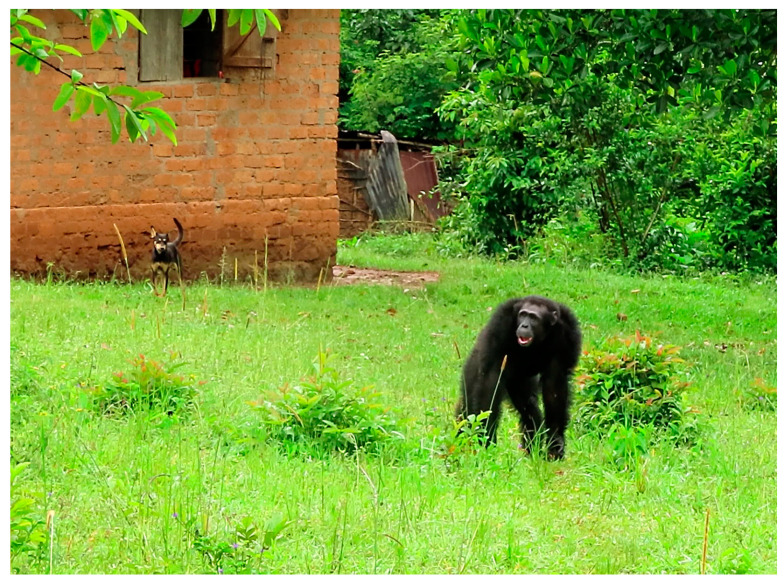
Close encounters between NHP, domestic animals, and humans are a daily occurrence in Bulindi. The image shows an adult male chimpanzee in proximity to a domestic dog in the compound of a village home.

The aim of this explorative study was to investigate *Giardia*, *Cryptosporidium*, and microsporidia infections for their potential zoonotic transmission among humans, domesticated animals (livestock, poultry and dogs), and wild NHP (eastern chimpanzees, *Pan troglodytes schweinfurthii*; and black and white colobus monkeys, *Colobus guereza*) in Bulindi, Uganda [52,53,54] (Figure 1) using immunochromatographic and molecular analyses.

## 2. Results

Two immunochromatographic assays were positive for the presence of *G. intestinalis* coproantigen of the 86 rapid tests performed, corresponding to a 2-year-old boy and a cow. Due to a limited number of tests available in the field, 86 fecal samples were tested for *G. intestinalis* and 137 for *Cryptosporidium*, out of the 137 feces collected. All the 137 *Cryptosporidium* immunochromatographic assays were negative (Table S1).

Out of 137 samples screened, specific DNA of *G. intestinalis* and *E. cuniculi* was detected in three and three samples, respectively (Table 1 and Table S1). Both samples that were positive for *G. intestinalis* by immunochromatographic assay were also positive by PCR. In addition, another sample, corresponding to an 8-year-old girl, was positive by PCR. Phylogenetic analysis of the TPI gene revealed the presence of *G. intestinalis* assemblage B in both human isolates, which were identical to isolate GenBank acc. no. EF688026, and *G. intestinalis* assemblage E in a cow isolate, which was identical to sequence GenBank acc. no. KJ363355 (Table 1). All three *ITS* sequences of *E. cuniculi* obtained from a 40-year-old man, a 2-year-old boy (from the same household), and a goat, were identical to *E. cuniculi* genotype II (e.g., GenBank acc. no. GQ422153), previously detected in a wide spectrum of hosts (Table 1). A mixed infection of *G. intestinalis* assemblage B and *E. cuniculi* genotype II was observed in a 2-year-old boy (Table 1 and Table S1). None of the screened human and animal samples were positive for the presence of specific DNA of *Cryptosporidium* spp. or *E. bieneusi*. None of the examined people or animals suffered from diarrhoea.

## 3. Discussion

A very low occurrence of *G. intestinalis* and *E. cuniculi*, and no occurrence of *Cryptosporidium* spp. and *E. bieneusi*, was detected in the present study and we found no evidence of potential transmission of the studied protists among closely coexisting people, domestic animals, and NHP in this farm–forest mosaic landscape in rural Uganda. Out of the *Giardia* assemblages identified in this study, only assemblage B, detected in two humans, has zoonotic potential, while assemblage E, which predominantly infects domestic ruminants and pigs (mainly cattle and sheep), causes human giardiosis only rarely [55]. The sequence of *G. intestinalis* assemblage B in this study was identical to isolates that have been previously reported from water samples and humans in Canada, humans in Australia (GenBank acc. no. EF688026), and from captive white-faced saki monkeys (*Pithecia pithecia*) in Japan [56,57]. *Encephalitozoon cuniculi* has a broad host range, mainly among mammals, but also infects birds, NHP, and humans [58]. Four different genotypes (I–IV) have so far been differentiated by analysis of the ITS region of ribosomal genes. Although there seems to be a certain host preference in each genotype, this specificity is not strict [59]. *Encephalitozoon cuniculi* genotype II, the most common genotype, has been reported in numerous birds and mammals, including livestock, NHP, and humans [58].

The results of our study contrast with several previous studies that reported a higher occurrence of the studied parasites in various primates with frequent contact with humans and livestock (e.g., eastern chimpanzees and baboons *P. anubis* [7]; long-tailed macaques *Macaca fascicularis* [60]; mountain gorillas *G. b. beringei* [61]). Rather, our findings are in accordance with studies reporting a low occurrence of *E. cuniculi*, *E. bieneusi*, or *Cryptosporidium* spp. in NHP [33,36] in areas with considerably less contact between NHP, humans, and livestock.

The infectious stages of the observed parasites, especially *G. intestinalis*, *E. bieneusi*, and *E. cuniculi*, are often excreted intermittently. Thus, repeated sampling of the same individuals over several consecutive days is recommended [62]. A low occurrence together with a limited number of samples can reduce the likelihood of parasite detection [26,63]. Nevertheless, in a previous coproscopic survey made at Bulindi in 2012–2013, McLennan et al. [53] collected 432 fecal samples from 19 chimpanzees and found a low prevalence of *Giardia* cysts (1.6%). These findings agree with the results of the present study and suggest a very low *G. intestinalis* burden in this population, even when approximatively 23 samples from each individual were analyzed. Our findings also reveal a very low occurrence of the other studied protists in humans and domesticated animals from Bulindi, but do not necessarily indicate that NHP in the study area are not at risk of *G. intestinalis* infections or other zoonotic pathogens (see, e.g., [54]). The low number of positive detections in this and previous studies could also be due to the very low infection rates of the studied pathogens. As has been shown, wild animals may harbor parasite infections at intensities under the detection limit of diagnostic methods. Generally, samples with an infection intensity of less than 500 and 10 (oo)cysts of *Cryptosporidium* spp. mostly failed in immunochromatographic assays and PCR, respectively [64,65,66]. Therefore, concentrating (oo)cysts in the sample, which is not often used in parasitological studies of NHP due to the small amount of available fecal material, could increase the detection rate of the studied protists.

Our findings suggest that humans and livestock in Bulindi also have low levels of protist infections compared to results of surveys elsewhere in Africa (e.g., [67,68,69,70,71,72,73]), including the only previous study using amplification by PCR and sequencing conducted in Uganda (around Kibale National Park, 150 km south of Bulindi). In that study, 40.7% of 108 human fecal samples were positive for *G. intestinalis* [31]. This difference may be linked to deworming treatments delivered to humans and domestic animals in the Bulindi area, in Hoima District. Adults (except pregnant women and children under 5 years) generally receive a periodical preventive onchocercosis treatment every six months with Ivermectin (treatments distributed by the Ugandan government) and, in parallel, children are dewormed every three months at their schools with albendazole (data collected during interviews by M. Cibot, unpubl. data). Most farmers also regularly treat their animals, except poultry, with albendazole (Albafas 25 mg, albendazole) at least once per year. Some also treat calves every month and adult cows every second month (Cibot, unpubl. data). We also cannot exclude a potential impact of improved sanitation among participating households; however, we were unable to compare sanitation practices in Bulindi with previous studies in other regions in the present study. Last but not least, the different sensitivity of the PCR methods used to detect *G. intestinalis* should be taken into account. While in our study we used genotyping at the locus encoding triosephosphate isomerase (tpi), Johnston et al. [31] used multilocus sequence typing at ef1-a (elongation factor 1), gdh (glutamate dehydro-genase), SSU (small subunit 18S rRNA), and tpi loci, which may increase the number of amplified positive samples for particular assemblages due to extensive annealing site diversity [74].

Albendazole is a broad spectrum antiparasitic agent that is also effective against giardiosis [75,76]. The positive effect of albendazole treatment for *G. intestinalis* infections in human and animal populations has been widely proven [77,78]. The limited and temporary effect of albendazole on *E. cuniculi* was reported under experimental conditions in both immunodeficient and immunocompetent murine hosts [79,80,81,82]. Moreover, in most immunocompetent hosts experimentally infected with *Encephalitozoon cuniculi*, treatment with albendazole caused a considerable shift of infection towards organs outside the gastrointestinal tract, disappearance of microsporidia from the gastrointestinal tract, and reduced spore shedding [80,81,82,83]. No 100% effective treatment is currently available to clear the infection caused by *Cryptosporidium* spp. Currently, nitazoxanide is used against cryptosporidiosis in immunocompetent patients and halofuginone lactate and paromomycin for livestock [84,85,86]. Albendazole and ivermectin are not standardly used for treatment of hosts suffering from cryptosporidiosis, but the effect of ivermectin against *C. parvum* infection was observed in a rat model under experimental conditions [87,88]. However, given the limited number of studies investigating the efficacy of ivermectin on *Cryptosporidium* infections, it cannot be stated with certainty that regular use of this drug in the Bulindi study population contributed to the absence of *Cryptosporidium* in the present study.

Repeated application of albendazole and ivermectin in Bulindi human residents and livestock, and potentially improved sanitation, probably guarantees long-term effects resulting in a minimum of positive samples and a decreasing potential circulation of these pathogens among host species. Similarly, a decreased prevalence of *Giardia* spp. in mountain gorillas in Uganda was related to improved health and sanitation among local humans [89]. While it is commonly assumed that close spatial overlap between humans and domestic or wild animals, including wild NHP, creates a high risk of pathogen transmission, the reality is likely to be more nuanced. As Narat et al. [15,90] point out, identifying how different kinds and frequencies of contact between species affect cross-transmission of pathogens, as well as different practices of health prevention, must be taken into account in parasitological studies.

To conclude, it is essential that long-term health monitoring of wild NHP, including the endangered chimpanzees [10], as well as humans and their domestic animals, is implemented in Bulindi and elsewhere regionally to better understand host and pathogen dynamics in such a dynamic, human-modified landscape where humans, livestock, and wildlife coexist closely.

## 4. Materials and Methods

Bulindi (1°29′ N, 31°28′ E) is located in western Uganda’s Hoima District. The landscape is a mosaic of farmland, villages, and fragments of riverine forest along watercourses. A resident ‘community’ of chimpanzees, first studied in 2006–2007 [91], has been studied continuously since 2014 [92]. Besides the chimpanzees, black and white colobus monkeys are also permanent NHP residents, whereas baboons (*Papio anubis*) and tantalus monkeys (*Chlorocebus tantalus*) are transient visitors. NHP in Bulindi have experienced major habitat disturbance: between 2006 and 2014 forest fragments were reduced in size by ca. 80% and converted to farmland [92]. Rapid habitat change has led to increased foraging in agricultural fields by NHP [91,92] and close encounters occur daily among the chimpanzees, black and white colobus monkeys, people, and domestic animals (Figure 1). Wild NHP defecate in croplands and near homes and dwellings when travelling or foraging outside forest fragments. Conversely, villagers use forest fragments for timber and fuelwood, and they also sometimes defecate outdoors at the edges of crop fields and in the forest [52]. Pigs, goats and cows are ordinarily kept near homes. However, cattle (sometimes with goats) are taken daily to graze along forest edges and to drink at forest streams. Dogs are usually free to roam. Finally, people, cattle, and NHP use shared water sources within forest fragments. Thus, risk of pathogen transmission in this landscape is extremely high [53,54].

During October–November 2016 (in the wet season [53]) we non-invasively collected fresh feces of chimpanzees (*n* = 30) and colobus monkeys (*n* = 17) inhabiting forest fragments in Bulindi. Chimpanzees (community size at the time of the study = 22 individuals) were followed daily by the field team and fresh fecal samples were picked immediately after defecation from identified individuals. Fresh fecal samples (estimated ≤12 h old) were collected from unhabituated black and white colobus monkeys from beneath trees where the monkeys had been located. In parallel, local human participants (*n* = 43), livestock (*n* = 11 cows, *n* = 11 goats, *n* = 12 pigs), poultry (*n* = 11), and dogs (*n* = 2) were sampled from 10 households in two villages located centrally within the 20 km^2^ home range of the chimpanzees (Table S1). We gave participants tongue placers, stool containers and plastic bags to enable them to collect samples by themselves, and returned a maximum of six hours later to collect them. For humans and domesticated animals, each fecal sample represented a unique individual sampled once only, but some individual chimpanzees were sampled more than once; colobus monkey individuals also could have been sampled more than once. The fecal consistency was noted at the time of sampling. The fecal specimens were preserved in 95% ethanol and shipped to the Institute of Parasitology, Czech Academy of Sciences. All samples were laboratory processed within 1–2 months after collection. Each human participant was also asked to participate in a short interview about their (and their children’s) (1) previous anthelmintic treatments and health status, and about their (2) animal husbandry practices (e.g., number of animals kept, housing for animals, medical treatments). As not all individuals in the study villages were literate and/or spoke English, a local translator helped with interviews.

*Giardia* Rapid Assay (IDEXX, Westbrook, ME, USA) and RIDA QUICK *Cryptosporidium* (R-Biopharm AG, Daemstadt, Germany) immunochromatographic diagnostic kits were used according to the directions of the manufacturer for detection of *Giardia* and *Cryptosporidium* coproantigen in fresh and ethanol fixed feces, respectively. RIDA QUICK *Cryptosporidium* is primarily developed for detection of *C. parvum*, and therefore other *Cryptosporidium* spp. might not be detected. The suspension of each fecal sample in ethanol was evaporated overnight at 60 °C, before isolation of genomic DNA (gDNA). A total of 200 mg of fecal material was homogenized by bead disruption using 0.5 mm glass beads (Biospec Products, Inc., Bartlesville, OK, USA) in a FastPrep^®^-24 Instrument (MP Biomedicals, CA, USA) at a speed of 5 m/s for 1 min followed by isolation/purification using the QIAamp^®^ DNA Stool Mini Kit in accordance with the manufacturer’s instructions (QIAgen, Hilden, Germany). Purified gDNA was stored at −20 °C prior to use in PCR. All gDNA samples obtained were analyzed by polymerase chain reaction (PCR) using sets of specific primers. A nested PCR approach was used to amplify a region of the triosephosphate isomerase gene (*TPI*) of *G. intestinalis* [93], small ribosomal subunit rRNA gene (*SSU*) of *Cryptosporidium* spp. [94], the internal transcribed spacer (*ITS*) of *Enterocytozoon bieneusi* [95] and *Encephalitozoon* spp. [26]. Molecular grade water and DNA of *Giardia microti*, *C. proliferans*, *E. hellem* genotype 1A, or *E. bieneusi* genotype PtEbIX were used as negative and positive controls, respectively. Secondary PCR products were run on a 2% agarose gel containing 0.2 µg/mL ethidium bromide in 1 × TAE buffer at 75 volts for approximately 1 h. Bands of the predicted size were visualised using an UV light source, and then extracted using QIAquick Gel Extraction Kit (QIAgen). Gel-purified secondary products were sequenced in both directions with an ABI 3130 genetic analyzer (Applied Biosystems, Foster City, CA, USA) using the secondary PCR primers and the BigDye Terminator V3.1 cycle sequencing kit (Applied Biosystems, Foster City, CA, USA). All samples were analyzed in duplicates. In the case of positive detection, the sample was newly re-isolated and the previous finding was independently verified. Sequences have been deposited in GenBank under the accession numbers MZ048410–MZ048412 (ITS of *Encephalitozoon cuniculi*) and MZ055371–MZ055373 (TPI of *Giardia intestinalis*).

## Figures and Tables

**Table 1:** Presence of *Giardia intestinalis* and *Encephalitozoon cuniculi* based on amplification of the triosephosphate isomerase gene (*TPI*) and the internal transcribed spacer (*ITS*) of the rRNA, respectively, by PCR in fecal samples (*n* = 137) of humans, domestic animals and NHP in Bulindi, Uganda. The asterisk (*) indicates samples that were positive for coproantigen by the immunochromatographic assay.

Host	Sex	Positive/No. of Screened Samples (Occurrence) [Family Origin]	Parasite Identification (GenBank Acc. No.)
Humans	M	2/19 (10.5%) [family 4]	*E. cuniculi* genotype II (MZ048410)
			*G. intestinalis* assemblage B * (MZ055371) *E. cuniculi* genotype II (MZ048411)
	F	1/24 (4.2%) [family 9]	*G. intestinalis* assemblage B (MZ055372)
Cows	ND	1/11 (9.1%) [family 3]	*G. intestinalis* assemblage E * (MZ055373)
Goats	ND	1/11 (9.1%) [family 9]	*E. cuniculi* genotype II (MZ048412)
Pigs	ND	0/12	–
Hens	ND	0/11	–
Dogs	ND	0/2	–
Chimpanzees	M	0/14	–
	F	0/16	–
Black and white colobus monkeys	ND	0/17	–

M—Male; F—Female; ND—Not determined.

## Data Availability

Data are contained within the article and supplementary material, and sequences generated in the study have been deposited in GenBank.

## Supplementary Materials

The following are available online at https://www.mdpi.com/article/10.3390/pathogens10080933/s1, Table S1: List of screened fecal samples (*n* = 137) from humans, domestic animals and NHP (chimpanzees and black and white colobus monkeys) in Bulindi, western Uganda; samples are listed by species and household membership for human participants and domestic animals (*n* = 10 households). Results of (1) immunochromatographic assays targeting coproantigen of *Giardia intestinalis* and *Cryptosporidium* spp.; and (2) presence/genotyping of specific DNA of *Cryptosporidium* spp., *Giardia intestinalis*, *Encephalitozoon* spp. and *Enterocytozoon bieneusi* based on amplification of the small ribosomal subunit rRNA gene (SSU), triosephosphate isomerase gene (TPI), and the internal transcribed spacer (ITS) of the rRNA, respectively, by PCR are shown. Positive samples are indicated by red.
