# Supplementary material for: Sparse Evidence for Giardia intestinalis, Cryptosporidium spp. and Microsporidia Infections in Humans, Domesticated Animals and Wild Nonhuman Primates Sharing a Farm–Forest Mosaic Landscape in Western Uganda
Source: Pathogens. 2021 Jul 23;10(8):933. doi: 10.3390/pathogens10080933 (PMC8398676; doi:10.3390/pathogens10080933)
Supplement: Supplementary file 1 [file pathogens-10-00933-s001.zip › pathogens-1234693-supplementary.pdf]

Table S1. List of screened fecal samples (n = 137) from humans, domestic animals and NHP (chimpanzees and black and white colobus monkeys) in Bulindi, western Uganda; samples are listed by species and household membership for human participants and domestic animals (n = 10 households). Results of (1) immunochromatographic assays targeting coproantigen of *Giardia intestinalis* and *Cryptosporidium* spp.; and (2) presence/genotyping of specific DNA of *Cryptosporidium* spp., *Giardia intestinalis, Encephalitozoon* spp. and *Enterocytozoon bieneusi* based on amplification of the small ribosomal subunit rRNA gene (SSU), triosephosphate isomerase gene (TPI), and the internal transcribed spacer (ITS) of the rRNA, respectively, by PCR are shown. Positive samples are indicated by red. NA – data not available; ND – immunochromatographic assay not done.

Sample number	Species	Sex	Age (yr)	Family	(1) Imunochromatographic assay		(2) Molecular analyses (targeted locus)			
					Giardia intestinalis	*Cryptosporidium* spp.	*Cryptosporidium* spp. (SSU)	*Giardia intestinalis* (TPI)	*Encephalitozoon* spp. (ITS)	*Enterocytozoon bieneusi* (ITS)
30797	Chimpanzee	M	7	NA	Negative	Negative	Negative	Negative	Negative	Negative
30804	Chimpanzee	M	7	NA	Negative	Negative	Negative	Negative	Negative	Negative
30810	Chimpanzee	M	7	NA	Negative	Negative	Negative	Negative	Negative	Negative
30805	Chimpanzee	F	NA	NA	Negative	Negative	Negative	Negative	Negative	Negative
30806	Chimpanzee	F	NA	NA	Negative	Negative	Negative	Negative	Negative	Negative
30807	Chimpanzee	F	NA	NA	Negative	Negative	Negative	Negative	Negative	Negative
30812	Chimpanzee	F	NA	NA	Negative	Negative	Negative	Negative	Negative	Negative
30817	Chimpanzee	F	NA	NA	Negative	Negative	Negative	Negative	Negative	Negative
30818	Chimpanzee	F	NA	NA	Negative	Negative	Negative	Negative	Negative	Negative
30794	Chimpanzee	M	7	NA	Negative	Negative	Negative	Negative	Negative	Negative
30820	Chimpanzee	M	7	NA	Negative	Negative	Negative	Negative	Negative	Negative
30802	Chimpanzee	F	3	NA	Negative	Negative	Negative	Negative	Negative	Negative
30819	Chimpanzee	M	8	NA	Negative	Negative	Negative	Negative	Negative	Negative
30821	Chimpanzee	F	12	NA	Negative	Negative	Negative	Negative	Negative	Negative
30798	Chimpanzee	M	8	NA	Negative	Negative	Negative	Negative	Negative	Negative
30796	Chimpanzee	F	12	NA	Negative	Negative	Negative	Negative	Negative	Negative
30809	Chimpanzee	F	9	NA	Negative	Negative	Negative	Negative	Negative	Negative
30811	Chimpanzee	F	9	NA	Negative	Negative	Negative	Negative	Negative	Negative
30801	Chimpanzee	F	39	NA	Negative	Negative	Negative	Negative	Negative	Negative
30799	Chimpanzee	F	17	NA	Negative	Negative	Negative	Negative	Negative	Negative
30808	Chimpanzee	F	17	NA	Negative	Negative	Negative	Negative	Negative	Negative
30793	Chimpanzee	F	0.5	NA	Negative	Negative	Negative	Negative	Negative	Negative
30813	Chimpanzee	M	2.5	NA	Negative	Negative	Negative	Negative	Negative	Negative
30795	Chimpanzee	M	12	NA	Negative	Negative	Negative	Negative	Negative	Negative
30800	Chimpanzee	M	12	NA	Negative	Negative	Negative	Negative	Negative	Negative
30803	Chimpanzee	M	27	NA	Negative	Negative	Negative	Negative	Negative	Negative
30815	Chimpanzee	M	27	NA	Negative	Negative	Negative	Negative	Negative	Negative
30822	Chimpanzee	M	27	NA	ND	Negative	Negative	Negative	Negative	Negative
30816	Chimpanzee	M	32	NA	Negative	Negative	Negative	Negative	Negative	Negative
30814	Chimpanzee	F	7	NA	Negative	Negative	Negative	Negative	Negative	Negative
30776	Black and white colobus monkey	NA	NA	NA	Negative	Negative	Negative	Negative	Negative	Negative
30777	Black and white colobus monkey	NA	NA	NA	Negative	Negative	Negative	Negative	Negative	Negative
30778	Black and white colobus monkey	NA	NA	NA	Negative	Negative	Negative	Negative	Negative	Negative
30779	Black and white colobus monkey	NA	NA	NA	Negative	Negative	Negative	Negative	Negative	Negative
30780	Black and white colobus monkey	NA	NA	NA	Negative	Negative	Negative	Negative	Negative	Negative
30781	Black and white colobus monkey	NA	NA	NA	Negative	Negative	Negative	Negative	Negative	Negative
30782	Black and white colobus monkey	NA	NA	NA	Negative	Negative	Negative	Negative	Negative	Negative
30783	Black and white colobus monkey	NA	NA	NA	Negative	Negative	Negative	Negative	Negative	Negative
30784	Black and white colobus monkey	NA	NA	NA	Negative	Negative	Negative	Negative	Negative	Negative
30785	Black and white colobus monkey	NA	NA	NA	Negative	Negative	Negative	Negative	Negative	Negative
30786	Black and white colobus monkey	NA	NA	NA	Negative	Negative	Negative	Negative	Negative	Negative
30787	Black and white colobus monkey	NA	NA	NA	ND	Negative	Negative	Negative	Negative	Negative
30788	Black and white colobus monkey	NA	NA	NA	ND	Negative	Negative	Negative	Negative	Negative
30789	Black and white colobus monkey	NA	NA	NA	ND	Negative	Negative	Negative	Negative	Negative
30790	Black and white colobus monkey	NA	NA	NA	ND	Negative	Negative	Negative	Negative	Negative
30791	Black and white colobus monkey	NA	NA	NA	ND	Negative	Negative	Negative	Negative	Negative
30792	Black and white colobus monkey	NA	NA	NA	ND	Negative	Negative	Negative	Negative	Negative
30834	Dog	NA	NA	1	Negative	Negative	Negative	Negative	Negative	Negative
30836	Goat	NA	NA	1	Negative	Negative	Negative	Negative	Negative	Negative
30837	Goat	NA	NA	1	Negative	Negative	Negative	Negative	Negative	Negative
30838	Goat	NA	NA	1	Negative	Negative	Negative	Negative	Negative	Negative
30839	Goat	NA	NA	1	Negative	Negative	Negative	Negative	Negative	Negative
30840	Goat	NA	NA	1	Negative	Negative	Negative	Negative	Negative	Negative
30841	Goat	NA	NA	1	Negative	Negative	Negative	Negative	Negative	Negative
30823	Hen	NA	NA	1	Negative	Negative	Negative	Negative	Negative	Negative
30731	Human	F	41	1	Negative	Negative	Negative	Negative	Negative	Negative
30732	Human	M	13	1	Negative	Negative	Negative	Negative	Negative	Negative
30733	Human	M	14	1	Negative	Negative	Negative	Negative	Negative	Negative
30734	Human	M	67	1	Negative	Negative	Negative	Negative	Negative	Negative
30858	Pig	NA	NA	1	Negative	Negative	Negative	Negative	Negative	Negative
30859	Pig	NA	NA	1	Negative	Negative	Negative	Negative	Negative	Negative
30735	Human	F	12	2	Negative	Negative	Negative	Negative	Negative	Negative
30736	Human	M	6	2	Negative	Negative	Negative	Negative	Negative	Negative
30847	Cow	NA	NA	3	Negative	Negative	Negative	Negative	Negative	Negative
30848	Cow	NA	NA	3	Positive	Negative	Negative	Assemblage E	Negative	Negative
30849	Cow	NA	NA	3	Negative	Negative	Negative	Negative	Negative	Negative
30850	Cow	NA	NA	3	Negative	Negative	Negative	Negative	Negative	Negative
30851	Cow	NA	NA	3	Negative	Negative	Negative	Negative	Negative	Negative
30826	Hen	NA	NA	3	Negative	Negative	Negative	Negative	Negative	Negative
30737	Human	M	10	3	Negative	Negative	Negative	Negative	Negative	Negative
30852	Cow	NA	NA	4	Negative	Negative	Negative	Negative	Negative	Negative
30853	Cow	NA	NA	4	Negative	Negative	Negative	Negative	Negative	Negative
30854	Cow	NA	NA	4	Negative	Negative	Negative	Negative	Negative	Negative
30824	Hen	NA	NA	4	Negative	Negative	Negative	Negative	Negative	Negative
30825	Hen	NA	NA	4	Negative	Negative	Negative	Negative	Negative	Negative
30738	Human	M	40	4	Negative	Negative	Negative	Negative	*E. cuniculi* genotype II	Negative
30739	Human	F	5	4	Negative	Negative	Negative	Negative	Negative	Negative
30740	Human	M	2	4	Positive	Negative	Negative	Assemblage B	*E. cuniculi* genotype II	Negative
30741	Human	F	34	4	Negative	Negative	Negative	Negative	Negative	Negative
30860	Pig	NA	NA	4	Negative	Negative	Negative	Negative	Negative	Negative
30835	Dog	NA	NA	5	Negative	Negative	Negative	Negative	Negative	Negative
30827	Hen	NA	NA	5	Negative	Negative	Negative	Negative	Negative	Negative
30828	Hen	NA	NA	5	Negative	Negative	Negative	Negative	Negative	Negative
30829	Hen	NA	NA	5	Negative	Negative	Negative	Negative	Negative	Negative
30830	Hen	NA	NA	5	Negative	Negative	Negative	Negative	Negative	Negative
30831	Hen	NA	NA	5	Negative	Negative	Negative	Negative	Negative	Negative
30742	Human	M	7	5	Negative	Negative	Negative	Negative	Negative	Negative
30743	Human	F	67	5	Negative	Negative	Negative	Negative	Negative	Negative
30744	Human	M	9	5	Negative	Negative	Negative	Negative	Negative	Negative
30745	Human	F	13	5	Negative	Negative	Negative	Negative	Negative	Negative
30861	Pig	NA	NA	5	Negative	Negative	Negative	Negative	Negative	Negative
30862	Pig	NA	NA	5	Negative	Negative	Negative	Negative	Negative	Negative
30863	Pig	NA	NA	5	Negative	Negative	Negative	Negative	Negative	Negative
30864	Pig	NA	NA	5	Negative	Negative	Negative	Negative	Negative	Negative
30865	Pig	NA	NA	5	Negative	Negative	Negative	Negative	Negative	Negative

30866	Pig	NA	NA	5	Negative	Negative	Negative	Negative	Negative	Negative
30746	Human	M	51	6	ND	Negative	Negative	Negative	Negative	Negative
30842	Goat	NA	NA	7	ND	Negative	Negative	Negative	Negative	Negative
30843	Goat	NA	NA	7	ND	Negative	Negative	Negative	Negative	Negative
30747	Human	M	33	7	ND	Negative	Negative	Negative	Negative	Negative
30748	Human	M	2,5	7	ND	Negative	Negative	Negative	Negative	Negative
30749	Human	F	8	7	ND	Negative	Negative	Negative	Negative	Negative
30750	Human	F	61	7	ND	Negative	Negative	Negative	Negative	Negative
30751	Human	F	9	7	ND	Negative	Negative	Negative	Negative	Negative
30752	Human	F	6	7	ND	Negative	Negative	Negative	Negative	Negative
30753	Human	F	29	7	ND	Negative	Negative	Negative	Negative	Negative
30754	Human	F	39	7	ND	Negative	Negative	Negative	Negative	Negative
30855	Cow	NA	NA	8	ND	Negative	Negative	Negative	Negative	Negative
30755	Human	F	11	8	ND	Negative	Negative	Negative	Negative	Negative
30756	Human	M	5	8	ND	Negative	Negative	Negative	Negative	Negative
30757	Human	F	36	8	ND	Negative	Negative	Negative	Negative	Negative
30758	Human	M	42	8	ND	Negative	Negative	Negative	Negative	Negative
30759	Human	M	10	8	ND	Negative	Negative	Negative	Negative	Negative
30760	Human	F	1,5	8	ND	Negative	Negative	Negative	Negative	Negative
30867	Pig	NA	NA	8	ND	Negative	Negative	Negative	Negative	Negative
30856	Cow	NA	NA	9	ND	Negative	Negative	Negative	Negative	Negative
30857	Cow	NA	NA	9	ND	Negative	Negative	Negative	Negative	Negative
30844	Goat	NA	NA	9	ND	Negative	Negative	Negative	Negative	Negative
30845	Goat	NA	NA	9	ND	Negative	Negative	Negative	*E. cuniculi* genotype II	Negative
30846	Goat	NA	NA	9	ND	Negative	Negative	Negative	Negative	Negative
30761	Human	F	34	9	ND	Negative	Negative	Negative	Negative	Negative
30762	Human	F	10	9	ND	Negative	Negative	Negative	Negative	Negative
30763	Human	F	8	9	ND	Negative	Negative	Assemblage B	Negative	Negative
30764	Human	M	6	9	ND	Negative	Negative	Negative	Negative	Negative
30765	Human	M	2,5	9	ND	Negative	Negative	Negative	Negative	Negative
30766	Human	F	14	9	ND	Negative	Negative	Negative	Negative	Negative
30767	Human	F	33	9	ND	Negative	Negative	Negative	Negative	Negative
30768	Human	M	10	9	ND	Negative	Negative	Negative	Negative	Negative
30868	Pig	NA	NA	9	ND	Negative	Negative	Negative	Negative	Negative
30869	Pig	NA	NA	9	ND	Negative	Negative	Negative	Negative	Negative
30833	Hen	NA	NA	10	ND	Negative	Negative	Negative	Negative	Negative
30832	Hen	NA	NA	10	ND	Negative	Negative	Negative	Negative	Negative
30771	Human	F	27	10	ND	Negative	Negative	Negative	Negative	Negative
30770	Human	F	9	10	ND	Negative	Negative	Negative	Negative	Negative
30769	Human	M	34	10	ND	Negative	Negative	Negative	Negative	Negative
30772	Human	F	22	10	ND	Negative	Negative	Negative	Negative	Negative
30773	Human	F	52	10	ND	Negative	Negative	Negative	Negative	Negative
